# Insomnia and Esketamine Add-On Therapy to Antidepressant Therapy in Patients with Treatment-Resistant Depression—A Pilot Study

**DOI:** 10.3390/ph18071066

**Published:** 2025-07-19

**Authors:** Daniel Szawarnoga, Joanna Fojcik, Michał Górski, Artur Pałasz, Marek Krzystanek

**Affiliations:** 1 Department of Psychiatry, Department of Neurology, Faculty of Health Sciences in Katowice, Medical University of Silesia in Katowice, 40-055 Katowice, Poland; daniel.szawarnoga@sum.edu.pl; 2 Department of Chronic Diseases and Civilization-Related Hazards, Faculty of Public Health in Bytom, Medical University of Silesia in Katowice, 40-055 Katowice, Poland; michal.gorski@sum.edu.pl; 3 Department of Histology, Faculty of Medical Sciences in Katowice, Medical University of Silesia, ul. Medyków 18, 40-752 Katowice, Poland; apalasz@sum.edu.pl; 4 Department and Clinic of Psychiatric Rehabilitation in Katowice, Faculty of Medical Sciences in Katowice, Medical University of Silesia in Katowice, 40-055 Katowice, Poland; mkrzystanek@sum.edu.pl

**Keywords:** esketamine, depression, treatment-resistant depression, insomnia, sleep disorders

## Abstract

**Background:** Insomnia, as one of the most common sleep disorders, is a significant health problem, especially among patients suffering from drug-resistant depression. Problems related to the quality of sleep in that population can significantly affect the effectiveness of treatment and quality of life, which is why it is necessary to search for effective therapeutic interventions in this area. Objective: The aim of this study was to compare the effectiveness of esketamine and other standard antidepressants in improving sleep quality in patients with drug-resistant depression. The main research question is whether and to what extent esketamine improves sleep parameters compared with other antidepressants. **Methods**: This study involves the analysis of data collected from 50 patients divided into two groups: those using esketamine in combination with other antidepressants and those using other antidepressants. The analysis of the results focuses on the assessment of differences in AIS scores between the groups assessed using the Athens Insomnia Scale (AIS). **Results:** Insomnia occurs much less frequently among people using esketamine than among people not using this drug. With the increase in the time of using esketamine and with the increase in the dose, the level of insomnia decreases. **Conclusions:** Esketamine brings about a rapid improvement in sleep quality, which is a significant advance in the treatment of drug-resistant depression. The obtained results not only confirm the effectiveness of esketamine but also show its advantage over traditional treatment methods in improving sleep quality.

## 1. Introduction

Treatment-resistant depression is a serious mental health disorder defined as the lack of response to at least two different antidepressant treatment regimens of appropriate dose and duration in the current episode of depression [1,2,3]. This condition is associated with persistent symptoms, including persistent low mood, loss of interest, sleep disturbances, and a sense of hopelessness, which significantly reduces the quality of life of patients [3].

In the treatment of depression, the main role is played by pharmacotherapy with antidepressants and psychotherapeutic interventions. Despite the availability of many different antidepressants, about 35% of patients still fail to achieve remission. In a certain group of patients, drug resistance (TRD) develops. The STAR-D study showed that about 30% of people diagnosed with depression during recurrent depressive disorders achieve remission after taking the first antidepressant in a given episode, and that remission rates decrease and relapse rates increase with the number of failed antidepressant treatments. Up to 15% of patients with depression suffer from drug-resistant depression, defined as a lack of response to treatment with two successive antidepressants from different groups, at the right dose and for the right duration [2,3]. Such patients are offered various pharmacological methods of treatment augmentation, which may be associated with systemic side effects (e.g., metabolic and sexual disorders) and limited efficacy.

Low remission rates in this group of patients underline the therapeutic difficulties and the need to search for more effective treatments, such as esketamine [1]. Furthermore, the treatment of drug-resistant depression requires intensive monitoring and significant resources, which generate high economic and social costs [3]. Upon analyzing the available data, it should be noted that the need to develop innovative therapies for this group of patients is particularly important, considering the limited benefits of traditional treatments.

Esketamine is an NMDA receptor antagonist and an agonist of the sigma-1 receptor. Many other mechanisms of action for esketamine have also been suggested, including its effect on second cellular signaling, epigenetic mechanisms, neuroplasticity, the opioid system, and nicotinic and muscarinic receptors [4,5]. Esketamine (S-ketamine), an enantiomer of ketamine, has recently gained acceptance as a modern drug used in the treatment of drug-resistant depression because of its rapid and significant antidepressant effect. Its mechanism of action makes it a promising solution in the treatment of cases in which traditional antidepressants are ineffective. At the same time, the effect of esketamine on sleep quality, although suggested in clinical studies, remains a subject that requires further investigation, especially in the aspect of comparing its effectiveness with standard methods of treating depression. For this reason, special attention is required to assess how esketamine affects various aspects of sleep, such as difficulty falling asleep, frequency of nocturnal awakenings, or subjective sleep quality [6,7].

## 2. Results

Data obtained from the analyzed studies are presented in detail in the tables below.

Women comprised the majority of participants in this study, constituting 56% of the respondents. The largest number of subjects (N = 15; 30%) were married, and a similar number (N = 12; 24%) were divorced. This study’s participants were mostly those who completed secondary education (60%) and who were gainfully employed (52%). The respondents did not differ statistically significantly in terms of the assessed characteristics of the study groups (detailed data are presented in Table 1).

In all patients in both groups, insomnia occurred with the same frequency. In both study groups—the one that took esketamine and the control group, which was not planned to take this drug—insomnia occurred to the greatest extent, i.e., above 10 points. The groups were homogeneous in this respect (Table 2).

The results indicated that in the second measurement, after a period of 6 months, the level of insomnia in patients treated with esketamine decreased significantly. After completion of this study, only one person from the group of 25 patients complained of insomnia, when the initial level of insomnia in this group was 100% (Table 3).

Insomnia occurs much less frequently among people using esketamine than among people not using this drug. The relationship is statistically significant (*p* < 0.001) and is characterized by a strong strength of the relationship (Kramer’s V = 0.961) (Table 4).

This study’s results show that in the group of patients using esketamine, the quality of sleep after 6 months of taking the drug improved significantly in a statistically significant manner (*p* = 0.016) (Table 5).

The results of this study show that in the group of patients who did not take esketamine, the quality of sleep after 6 months remained at a similar level compared with the initial level. The statistical analysis did not show statistically significant differences (Table 6).

The results of this study show that in the group of patients using esketamine, after a period of 6 months of taking the drug, the well-being of patients the next day after the night is significantly improved. These differences are statistically significant (Table 7).

The results of this study show that in the group of patients who did not take esketamine, the well-being on the next day in the measurement after 6 months remains at a similar level in relation to the initial level. No statistically significant differences were found in this group of patients after re-evaluation (Table 8).

The results of this study show that in the group of patients using esketamine, after 6 months of taking the drug, mental and physical performance significantly improved the next day (*p* < 0.001) (Table 9).

The results of this study show that in the group of patients who did not take esketamine, mental and physical performance on the next day, measured after 6 months, remains at a similar level compared with the initial level (*p* = 0.62) (Table 10).

With the increase in the time of esketamine use and with the increase in the dose, the level of insomnia decreases according to the Athens Insomnia Scale. The correlations are statistically significant (*p* < 0.001) and are characterized by strong strengths of association determined by the Spearman rho coefficient (Table 11).

## 3. Discussion

Treatment-resistant depression poses numerous challenges for researchers, both diagnostic and therapeutic. Developing effective treatment strategies that include both improving depressive symptoms and sleep quality is key to improving the overall condition of patients.

Patients with drug-resistant depression often report sleep problems, including insomnia, restless sleep, or early awakening, which have a significant impact on their overall quality of life and treatment effectiveness [5]. Sleep disorders can lead to the exacerbation of depressive symptoms and, at the same time, hinder the body’s regeneration [7]. Problems such as nocturnal awakening or early awakening indicate the need for treatment modulating the circadian rhythm, which can be achieved with innovative therapies such as esketamine [3].

Insomnia, which often coexists with drug-resistant depression, is one of the key aspects influencing the effectiveness of treatment. Esketamine, through its unique mechanisms of action on NMDA receptors, shows the potential to reduce nighttime wakefulness and improve the overall quality of sleep, which has a positive effect on the clinical condition of patients [1,2,3,4].

Comparing the efficacy of esketamine to standard antidepressants is crucial to understanding how different therapies can affect the treatment of treatment-resistant depression and improve the quality of sleep in patients. While traditional antidepressants such as escitalopram or aripiprazole show limited effects on sleep structure and architecture, esketamine offers both faster and more noticeable effects in this area [8,9].

Esketamine, acting as a modulator of NMDA receptors, affects specific sleep parameters, visible as a shorter time needed to fall asleep and a reduced number of nocturnal awakenings. This effect is a direct result of esketamine’s action on NMDA receptors, which stimulates neuronal plasticity and stabilizes neurotransmitter activity responsible for sleep regulation [6]. Compared with SSRIs, which often cause negative changes in the REM sleep architecture, esketamine has a more comprehensive effect on sleep cycles, which is important from the point of view of body regeneration. This is confirmed by the results of this study, which showed that insomnia is very rarely a problem in patients taking esketamine. Of the twenty-six patients from both groups reporting insomnia, only one person was from the group using esketamine. This result is extremely satisfactory and confirms the importance of this drug in the treatment of drug-resistant depression and insomnia occurring with it.

Subjective patient assessments, measured using the Athens Insomnia Scale (AIS), confirm that esketamine brings rapid and noticeable effects within the first weeks of therapy, which distinguishes it from standard therapies that require a longer period of use to obtain similar results [6].

Although standard antidepressants such as escitalopram and aripiprazole are effective for long-term reductions in depressive symptoms, their effects on sleep quality are more limited compared with esketamine. Ahuja et al. noted that traditional medications can lead to chronic daytime sleepiness, which negatively affects the quality of life of patients [10,11,12]. In contrast, esketamine is well tolerated, and its side effects, such as mild headaches or nausea, are transient and do not interfere with sleep parameters [6,13]. Moreover, esketamine has a beneficial effect on the regulation of circadian rhythms, which may improve the overall functioning of patients during the day [14]. Healthy sleep–wake rhythms are crucial in the treatment of treatment-resistant depression, where the psychological and physiological effects of sleep disorders often overlap with depressive symptoms [6].

Improved sleep as an integral part of the healing process is an additional therapeutic benefit that distinguishes esketamine from traditional antidepressants. Studies conducted by Lal and Son indicate that better sleep quality correlates with a reduction in the severity of depressive symptoms, which suggests that esketamine may also support the improvement of patients’ functioning in daily life [1,2,3,4,5,6,7]. Additionally, studies conducted by Italian scientists indicate that insomnia was a risk factor for suicide among adolescent psychiatric patients, which is extremely important information from a clinical point of view [15].

The results showed that esketamine, thanks to its unique mechanism of action on NMDA receptors, can bring about rapid improvement in sleep quality, which is a significant advance in the treatment of drug-resistant depression. The results obtained not only confirm the effectiveness of esketamine but also show its advantage over traditional methods of treatment in terms of improving sleep quality.

The conducted study significantly enriches the existing knowledge by showing the specific therapeutic potential of esketamine in the treatment of drug-resistant depression, especially in the context of sleep disorders. Previous studies on standard antidepressants have often indicated their limited effect on sleep quality, and even the possibility of deterioration of their architecture, especially REM sleep. Esketamine, thanks to the modulation of NMDA and possibly AMPA receptors and the improvement of neuronal plasticity, is an innovative pharmacological option that simultaneously reduces depressive symptoms and improves sleep parameters. The presented results confirm earlier reports indicating that subanesthetic doses of racemic ketamine significantly promoted wakefulness [16,17]. Further studies revealed that a single administration of esketamine enhanced wakefulness while increasing NREM sleep latency (NREM sleep latency) in rats [18,19]. The neuromolecular mechanism of this effect and the involvement of specific neurotransmitters are not clear, but NMDA receptors undoubtedly play a key role. It is worth emphasizing that esketamine is characterized by over four times stronger affinity for NMDA receptors than erketamine (R-ketamine) [20]. The use of CPP, a selective NMDAR antagonist, in an animal model increased the release of dopamine in the prefrontal cortex (PFC), which resulted in the strengthening of the arousal state and increased motor activity [21]. The effect of esketamine, but not erketamine, also resulted in an increase in the release of this neurotransmitter in the PFC. Both enantiomers of ketamine increased the level of noradrenaline in this brain region [22]. It is also possible that esketamine affects NMDAR-dependent glutamatergic signaling at the hypothalamus [23], especially its lateral preoptic area (LPO). NMDAR-expressing neurons in this region are responsible for maintaining NREM and REM sleep [24]. Extrasynaptic, long-open NMDARs can stabilize hypothalamic sleep-on neurons [25]. It is also worth noting that the blockade of lateral habenula neurons caused significant fragmentation of NREM sleep and wakefulness in mice with unchanged total duration of both phases [26]. The disruption of NMDAR function in these neurons can also result in insomnia, suggesting that the receptors are required for the firing of neurons in the lateral habenula [27].

Considering the importance of sleep in regulating emotions and reducing depressive symptoms, it is necessary to implement therapeutic strategies aimed at improving its quality [3]. However, it is worth analyzing the need to integrate pharmacological therapy with non-pharmacological support methods that could contribute to a better therapeutic effect and affect the quality of life of patients, which is important in the treatment of drug-resistant depression. It is also worth considering the implementation of future research directions aimed at more detailed analyses of the group of studied patients (larger sample size, subgroup analyses, etc.), which can describe the research topic in more detail.

Even though the cost of esketamine treatment is high, the demonstrated effectiveness of the drug in reducing insomnia is very important from a clinical point of view. Esketamine treatment is therefore possible with the introduction of drug reimbursement or because of an individual patient’s decision after the doctor presents various therapeutic options.

The authors of this study know the group size is relatively small to allow for a reliable multivariate analysis, but the results of this study were so interesting that the authors decided to publish them. Due to the small group size, the authors also did not analyze the groups studied in terms of the severity of depression, nutrition, hormonal phase, and health status.

Although AIS is a valid measure of insomnia, the reliance on a subjective self-report questionnaire limits the objectivity of the results. The conclusions of this study are also limited because, obviously, the observed correlation between treatment time and effect on insomnia does not imply a causal relationship.

## 4. Materials and Methods

This study was conducted at the Mental Health Center in Bytom among patients with drug-resistant depression who were subjected to pharmacotherapy with esketamine in combination with SSRI antidepressants and SSRIs alone. The Athens Insomnia Scale (Appendix A) and our own demographic and clinical survey were used for this study.

Patients were divided into two groups. One group consisted of patients taking esketamine in combination with SSRI antidepressants; this group was named “using esketamine.” The other group consisted of patients not taking esketamine but taking antidepressants from the SSRI group; this group was called “not using esketamine” for the purposes of this study.

Insomnia was measured twice in both study groups: the first measurement was taken before this study began, and the second measurement after 6 months. The study group using esketamine received esketamine (dose up to 84 mg/day) within 6 months of the first insomnia measurement.

The group of patients not taking esketamine used fluoxetine, paroxetine, or sertraline. In contrast, the group of patients taking esketamine used fluoxetine or sertraline. In both groups, patients did not take any other medications that could affect sleep. Patients taking SSRIs had been taking them for at least 12 weeks without any improvement in depressive symptoms.

### 4.1. Statistical Analysis

The statistical analysis was performed using the IBM SPSS 26.0 package with the Exact Tests module. All relationships, correlations, and differences are statistically significant when *p* ≤ 0.05. Correlations between ordinal or quantitative variables (when the conditions for using parametric tests were not met) were performed using Spearman’s rho coefficient, which provides information about the intensity of the relationship and its direction—positive or negative. The obtained value ranges from −1 to 1, where (−1) means a perfect negative correlation and (1) a perfect positive correlation [28,29]. The minimum group size was calculated using the G-power program.

Chi-square for the independence of variables was used mainly for questions built on nominal scales. To determine the strength of the relationship, coefficients based on the test were used: Phi and Kramer’s V. For each analysis with the Chi-square test, additional tests were performed, which are conducted especially in small samples. These are tests performed using the following methods: exact or Monte Carlo. Under each crosstabulation, the Chi-square test result is accompanied by the letter (a)—it means that the calculated statistic may not meet the condition of the minimum expected number; therefore, for this eventuality, the exact or Monte Carlo method is also used. In this case, if the “*p*” value is calculated based on the Monte Carlo method, it is additionally marked with the letter (b). The significance of “*p*” of the Phi and Kramer’s V coefficients is determined based on the Chi-square test result [10,30]. Due to the small number of observations in some categories of the contingency table (less than 5 cases), Fisher’s exact test was used to analyze the significance of differences in selected characteristics between the group using esketamine and the group not using esketamine.

### 4.2. AIS

The Athens Insomnia Scale (AIS) is a valuable research and diagnostic tool designed specifically to accurately assess the severity of insomnia symptoms. The scale includes eight key questions regarding various aspects of sleep quality, such as difficulty falling asleep, frequency of awakenings, sleep duration, and overall subjective assessment of sleep satisfaction.

The AIS is characterized by high reliability and psychometric validity, which is confirmed by numerous clinical studies on sleep quality in various populations, including patients with drug-resistant depression [1,2,3]. The validity of the AIS allows for precise differentiation between different levels of sleep disorder severity, which is crucial for an accurate assessment of patients’ conditions in clinical studies. This tool has been tested in numerous contexts, such as patients with PTSD or depressive disorders, which makes it universal and effective in various clinical environments [1,2,3]. The high reliability of the scale guarantees the repeatability of results, which is crucial in studies on the effect of therapies such as esketamine on sleep quality [1,2].

### 4.3. Information on the Populations Covered by This Study

A total of 50 patients (28 women and 22 men) diagnosed with treatment-resistant depression, aged 20 to over 50 years, were qualified for the clinical study. The inclusion criteria in this study’s groups were a diagnosis of treatment-resistant depression in patients and active pharmacotherapy with SSRI drugs. For all patients participating in this study, the inclusion criteria in the individual groups also included obtaining informed consent to this study, and the patient’s health condition preventing them from completing the questionnaires. The exclusion criteria from this study’s groups were the diagnosis of depression other than drug-resistant depression in patients and active pharmacotherapy with antidepressant drugs other than SSRIs.

According to the recommendation of the Polish Psychiatric Association, in order to qualify for esketamine treatment, the patient must meet, among others, the following criteria: confirmation of drug-resistant depression defined as depressive disorders in adults who have not responded to at least two different antidepressants (used at an appropriate dose for an appropriate period of time, according to the guidelines of the Polish Psychiatric Association) in the current episode of moderate-to-severe depression and at least two, no more than five different antidepressants in the current episode of depression. The standard of treatment with esketamine does not assume the use of only this drug without combination with other drugs, which explains the fact that there is no study group that takes only esketamine. This study was approved by the Bioethics Committee No. BNW/NWN/0052/KB/104/25.

## 5. Conclusions

Patients with treatment-resistant depression treated with esketamine suffer from insomnia less often than patients who do not take it, and the severity of sleep disorders decreases with time of treatment.

## Figures and Tables

**Table 1:** Characteristics of the study groups divided into groups using esketamine (N = 25) and not using esketamine (N = 25).

Variable	Total N; %	Group		*p*-Value *
		Using Esketamine N; %	Not Using Esketamine N; %	
Age (years)				
18–25	5; 10%	2; 8%	3; 12%	0.35 *
26–35	14; 28%	8; 32%	6; 24%	
36–45	14; 28%	8; 32%	6; 24%	
46–60	11; 22%	5; 20%	6; 24%	
60–75	6; 12%	2; 8%	4; 16%	
Sex				
Woman	28; 56%	13; 52%	15; 60%	0.73 *
Man	22; 44%	12; 48%	10; 40%	
Marital status				
Bachelor/Single	11; 22%	7; 28%	4; 16%	0.42 *
Marriage	15; 30%	9; 36%	6; 24%	
Civil partnership (informal)	4; 8%	1; 4%	3; 12%	
Divorced	12; 24%	4; 16%	8; 32%	
Widower/widow	8; 16%	4; 16%	4; 16%	
Education				
Basic	7; 14%	2; 8%	5; 20%	0.38 *
Medium	30; 60%	14; 56%	16; 64%	
Higher	13; 26%	9; 36%	4; 16%	
Professional status				
Working	26; 52%	16; 64%	10; 40%	0.12 *
Unemployed	15; 30%	6; 24%	9; 36%	
Pensioner	2; 4%	0	2; 8%	
Pupil/student	7; 14%	3; 12%	4; 16%	
Domicile				
City	15; 30%	9; 36%	6; 24%	0.33 *
Village	35; 70%	16; 64%	19; 76%	

* χ^2^ test.

**Table 2:** The occurrence of insomnia in individual groups—baseline measurement.

		Control Group		Study Group	
		N	%	N	%
Athens Insomnia Scale (AIS)	no insomnia (0–5)	0	0.0%	0	0.0%
	single symptoms of insomnia (6–10)	0	0.0%	0	0.0%
	insomnia (over 10)	25	100.0%	25	100.0%
	Total	25	100.0%	25	100.0%

**Table 3:** The occurrence of insomnia in individual groups—measurement after 6 months.

		Control Group		Study Group	
		N	%	N	%
Athens Insomnia Scale (AIS)	no insomnia (0–5)	19	76.0%	0	0%
	single symptoms of insomnia (6–10)	5	20.0%	0	0%
	insomnia (over 10)	1	4.0%	25	100%
	Total	25	100.0%	25	100%

**Table 4:** The occurrence of insomnia in individual groups—comparison of groups after 6 months.

			Use of Esketamine		Total
			Control Group	Study Group	
Athens Insomnia Scale AIS	no insomnia (0–5)	N	0	19	19
		%	0.0%	76.0%	38.0%
	single symptoms of insomnia (6–10)	N	0	5	5
		%	0.0%	20.0%	10.0%
	insomnia (over 10)	N	25	1	26
		%	100.0%	4.0%	52.0%
Total		N	25	25	50
		%	100.0%	100.0%	100.0%
V Cramer	0.961	46,154	2	0.000	0.000
factor	value	Chi-square	df	*p*	p Monte Carlo

**Table 5:** Sleep quality assessment in the study group.

Athens Insomnia Scale (AIS)		Output Level		After 6 Months		*p* *
		N	%	N	%	
Sleep quality	satisfactory	0	0.0%	12	48.0%	0.016
	slightly unsatisfactory	0	0.0%	12	48.0%	
	clearly unsatisfactory	15	60.0%	1	4.0%	
	completely unsatisfactory	10	40.0%	0	0.0%	
	Total	25	100.0%	25	100.0%	

* Fisher’s test.

**Table 6:** Sleep quality assessment in the control group.

Athens Insomnia Scale (AIS)		Output Level		After 6 Months		*p* *
		N	%	N	%	
Sleep quality	satisfactory	0	0.0%	0	0.0%	0.21
	slightly unsatisfactory	0	0.0%	4	16.0%	
	clearly unsatisfactory	12	48.0%	13	52.0%	
	completely unsatisfactory	13	52.0%	8	32.0%	
	Total	25	100.0%	25	100.0%	

* Fisher’s test.

**Table 7:** Assessment of well-being the next day for patients in the study group.

Athens Insomnia Scale (AIS)		Output Level		After 6 Months		*p* *
		N	%	N	%	
How I feel the next day	Good	0	0.0%	17	68.0%	0.003
	slightly worse	0	0.0%	8	32.0%	
	clearly worse	14	56.0%	0	0.0%	
	significantly worse	11	44.0%	0	0.0%	
	Total	25	100.0%	25	100.0%	

* Fisher’s test.

**Table 8:** Assessment of well-being the next day of patients in the control group.

Athens Insomnia Scale (AIS)		Output Level		After 6 Months		*p* *
		N	%	N	%	
How I feel the next day	Good	0	0.0%	0	0.0%	0.31
	slightly worse	1	4.0%	5	20.0%	
	clearly worse	9	36.0%	7	28.0%	
	significantly worse	15	60.0%	13	52.0%	
	Total	25	100.0%	25	100.0%	

* Fisher’s test.

**Table 9:** Assessment of mental and physical fitness on the next day in the study group.

Athens Insomnia Scale (AIS)		Output Level		After 6 Months		*p* *
		N	%	N	%	
Mental and physical fitness the next day	undisturbed	0	0.0%	16	64.0%	<0.001
	slightly disturbed	0	0.0%	9	36.0%	
	clearly disturbed	15	60.0%	0	0.0%	
	extremely disturbed	10	40.0%	0	0.0%	
	Total	25	100.0%	25	100.0%	

* Fisher’s test.

**Table 10:** Assessment of mental and physical fitness on the next day in the control group.

Athens Insomnia Scale (AIS)		Output Level		After 6 Months		*p* *
		N	%	N	%	
Mental and physical fitness the next day	undisturbed	0	0.0%	0	0.0%	0.62
	slightly disturbed	0	0.0%	0	0.0%	
	clearly disturbed	13	52.0%	11	44.0%	
	extremely disturbed	12	48.0%	14	56.0%	
	Total	25	100.0%	25	100.0%	

* Fisher’s test.

**Table 11:** Duration of esketamine use and its dose.

			How Long Have You Been Using Esketamine?	Esketamine Dose
Spearman’s rho	Athens Insomnia Scale AIS	Correlation coefficient	−0.711 **	−0.854 **
		Significance (bilateral)	0.000	0.000
		N	50	50

** correlation significant at the 0.01 level (bilateral).

## Data Availability

The original contributions presented in this study are included in the article. Further inquiries can be directed to the corresponding authors.

## Appendix A. No. 1 Athens Insomnia Scale (AIS)

………….. gender M/F age…………. Date of examination ………………………………

Please respond to each statement by marking the number on a scale from 1 to 5 that best describes your current state. Your choice should be indicated by circling the appropriate number. Each answer is correct as long as it is true.

1. I have trouble adapting to the limitations imposed by the disease. Definitely Definitely I agree 1 2 3 4 5 I don’t agree

2. Due to my health condition, I am not able to do what I like the most. Definitely Definitely I agree 1 2 3 4 5 I don’t agree

3. The disease sometimes makes me feel useless. Definitely Definitely I agree 1 2 3 4 5 I don’t agree

4. Health problems make me more dependent on others than I want to be Definitely Definitely I agree 1 2 3 4 5 I don’t agree

5. My illness makes me a burden to my family and friends. Definitely Definitely I agree 1 2 3 4 5 I don’t agree

6. My health condition makes me feel like I am not a fully-fledged person. Definitely Definitely I agree 1 2 3 4 5 I don’t agree

7. I will never be as self-sufficient as I would like to be. Definitely Definitely I agree 1 2 3 4 5 I don’t agree

8. I think that people around me are often embarrassed because of my illness. Definitely Definitely I agree 1 2 3 4 5 I don’t agree

AKC

…………………………………………………………………………

## References

[1] Price M.Z., Price R.L. (2024). Benefits and risks of esketamine nasal spray continuation in treatment-resistant depression. Biomark. Neuropsychiatry.

[2] Kwaśny A., Włodarczyk A., Ogonowski D., Cubała W.J. (2023). Effect of Ketamine on Sleep in Treatment-Resistant Depression: A Systematic Review. Pharmaceuticals.

[3] Rodrigues N.B., McIntyre R.S., Lipsitz O., Cha D.S., Cao B., Lee Y., Gill H., Lui L.M.W., Cubała W.J., Ho R. (2022). Do sleep changes mediate the anti-depressive and anti-suicidal response of intravenous ketamine in treatment-resistant depression?. J. Sleep Res..

[4] McIntyre R.S., Jain R. (2024). Glutamatergic Modulators for Major Depression from Theory to Clinical Use. CNS Drugs.

[5] Levinstein M.R., Budinich R.C., Bonaventura J., Schatzberg A.F., Zarate C.A., Michaelides M. (2025). Redefining Ketamine Pharmacology for Antidepressant Action: Synergistic NMDA and Opioid Receptor Interactions?. Am. J. Psychiatry.

[6] Kim J., Lee S.-H., Shin C., Han K.-M., Cho S.J., Hong N., Han C. (2024). A multi-center, open-label, single-arm study to investigate the early effectiveness of esketamine nasal spray in patients with treatment-resistant depression using a mobile self-monitoring application. Pharmaceuticals.

[7] Sun L., Zhao Y., Li Y., Zhai W., Gao F., Yin Q., Cheng W., Wang Z., Zeng Y. (2023). Effect of continuous subanesthetic esketamine infusion on postoperative fatigue. Am. J. Cancer Res..

[8] Ahuja S., Brendle M., Smart L., Moore C., Thielking P. (2023). Real-world patient characteristics, treatment patterns, efficacy and safety of intramuscular ketamine treatment. BMC Psychiatry.

[9] Mansuri Z., Shah B., Yadav G., Kamil S.H., Kainth T., Srinivas S., Patel H., Reddy A., Bachu A. (2024). Is Intravenous Ketamine Better Than Intranasal Esketamine for Treating Treatment-Resistant Depression?. Prim. Care Companion CNS Disord..

[10] Szymczak W. (2008). Podstawy Statystyki Dla Psychologów.

[11] Qiu D., Wang X.-M., Yang J.-J., Chen S., Yue C.-B., Hashimoto K., Yang J.-J. (2022). Effect of intraoperative esketamine infusion on postoperative sleep disturbance after gynecological laparoscopy. JAMA Netw. Open.

[12] Yan R., Marshall T., Khullar A., Nagle T., Knowles J., Malkin M., Chubbs B., Swainson J. (2024). Patient-reported outcomes on sleep quality and circadian rhythm during treatment with intravenous ketamine for treatment-resistant depression. Ther. Adv. Psychopharmacol..

[13] Cichocka A., Rozenek E.B., Michałowska K., Szewczak B., Waszkiewicz N. (2021). The importance of esketamine in the treatment of treatment-resistant depression. Will it prove to be a breakthrough drug?. Psychiatry.

[14] McIntyre R.S., Rosenblat J.D., Nemeroff C.B., Sanacora G., Murrough J.W., Berk M., Brietzke E., Stahl S. (2021). Synthesizing the evidence for ketamine and esketamine in treatment-resistant depression. Am. J. Psychiatry.

[15] Baldini V., Gnazzo M., Maragno M., Biagetti R., Stefanini C., Canulli F., Varallo G., Donati C., Neri G., Fiorillo A. (2025). Risk of suicide among adolescent psychiatric patients: The role of insomnia, depression, and socio-personal factors. Eur. Psychiatry.

[16] Ahnaou A., Huysmans H., Biermans R., Manyakov N.V., Drinkenburg W.H.I.M. (2017). Ketamine: Differential neurophysiological dynamics in functional networks in the rat brain. Transl. Psychiatry..

[17] Banerjee P., Donello J.E., Hare B., Duman R.S. (2020). Rapastinel, an NMDAR positive modulator, produces distinct behavioral, sleep, and EEG profiles compared with ketamine. Behav. Brain Res..

[18] Raith H., Schuelert N., Duveau V., Roucard C., Plano A., Dorner-Ciossek C., Ferger B. (2020). Differential effects of traxoprodil and S-ketamine on quantitative EEG and auditory event-related potentials as translational biomarkers in preclinical trials in rats and mice. Neuropharmacology.

[19] Koncz S., Papp N., Pothorszki D., Bagdy G. (2023). (S)-Ketamine but Not (R)-Ketamine Shows Acute Effects on Depression-Like Behavior and Sleep-Wake Architecture in Rats. Int. J. Neuropsychopharmacol..

[20] Scotton E., Antqueviezc B., Vasconcelos M.F., Dalpiaz G., Paul Géa L., Ferraz Goularte J., Colombo R., Ribeiro Rosa A. (2022). Is (R)-ketamine a potential therapeutic agent for treatment-resistant depression with less detrimental side effects? A review of molecular mechanisms underlying ketamine and its enantiomers. Biochem. Pharmacol..

[21] Del Arco A., Segovia G., Mora F. (2008). Blockade of NMDA receptors in the prefrontal cortex increases dopamine and acetylcholine release in the nucleus accumbens and motor activity. Psychopharmacology.

[22] Ago Y., Tanabe W., Higuchi M., Tsukada S., Tanaka T., Yamaguchi T., Igarashi H., Yokoyama R., Seiriki K., Kasai A. (2019). (R)-Ketamine induces a greater increase in prefrontal 5-HT release than (S)-ketamine and ketamine metabolites via an AMPA receptor-independent mechanism. Int. J. Neuropsychopharmacol..

[23] Miracca G., Anuncibay-Soto B., Tossell K., Yustos R., Vyssotski A.L., Franks N.P., Wisden W. (2022). NMDA Receptors in the Lateral Preoptic Hypothalamus Are Essential for Sustaining NREM and REM Sleep. J. Neurosci..

[24] Paoletti P., Bellone C., Zhou Q. (2013). NMDA receptor subunit diversity: Impact on receptor properties, synaptic plasticity and disease. Nat. Rev. Neurosci..

[25] Neupane C., Sharma R., Pai Y.H., Lee S.Y., Jeon B.H., Kim H.W., Stern J.E., Park J.B. (2021). High salt intake recruits tonic activation of NR2D subunit-containing extrasynaptic NMDARs in vasopressin neurons. J. Neurosci..

[26] Gelegen C., Miracca G., Ran M.Z.Z., Harding E.C., Ye Z.W., Yu X., Tossell K., Houston C.M., Yustos R., Hawkins E.D. (2018). Excitatory pathways from the lateral habenula enable propofol-induced sedation. Curr. Biol..

[27] Cui Y., Hu S., Hu H. (2019). Lateral habenular burst firing as a target of the rapid antidepressant effects of ketamine. Trends Neurosci..

[28] Górniak J., Wachnicki J. (2008). First Steps in Data Analysis.

[29] Nawojczyk M. (2002). Guide to Statistics for Sociologists.

[30] Bedyńska S., Brzezicka A. (2007). Statistical Signpost.

